# Mutant p53 upregulates HDAC6 to resist ER stress and facilitates Ku70 deacetylation, which prevents its degradation and mitigates DNA damage in colon cancer cells

**DOI:** 10.1038/s41420-025-02433-9

**Published:** 2025-04-10

**Authors:** Rossella Benedetti, Michele Di Crosta, Maria Saveria Gilardini Montani, Gabriella D’Orazi, Mara Cirone

**Affiliations:** 1 Department of Experimental Medicine, “Sapienza” University of Rome, Rome, Italy; 2 UniCamillus—Saint Camillus International University of Health and Medical Sciences, Rome, Italy; 3 Unit of Cellular Networks and Molecular Therapeutic Targets, IRCCS Regina Elena National Cancer Institute, Rome, Italy

**Keywords:** Post-translational modifications, Colorectal cancer

## Abstract

Cancer cells employ interconnected mechanisms to withstand intrinsic and extrinsic stress, with mutant p53 (mutp53) playing a key role in bolstering resistance to endoplasmic reticulum (ER) stress. In this study, we further investigated this phenomenon, focusing on the DNA damage triggered by ER stress. Our findings indicate that mutp53 mitigates ER stress-induced DNA damage by sustaining high levels of Ku70, a critical protein in DNA repair via the non-homologous end joining (NHEJ) pathway, which functions alongside Ku80. HDAC6 upregulation emerged as a crucial driver of this response. HDAC6 deacetylates Ku70, promoting its nuclear localization and protecting it from degradation. This mechanism ensures continuous activity of the NHEJ repair pathway, allowing mutp53-expressing cells to better manage DNA damage from ER stress, thus contributing to the genomic instability characteristic of cancer progression. Furthermore, HDAC6 maintains the activation of the ATF6 branch of the unfolded protein response (UPR), enhancing the ability of mutp53 cells to resist ER stress, as ATF6 supports cellular adaptation to misfolded proteins and stressful conditions. Since HDAC6 is central to this enhanced stress resistance and DNA repair, targeting it could disrupt these protective mechanisms, increasing the vulnerability of mutp53 cancer cells to ER stress and inhibiting cancer progression.

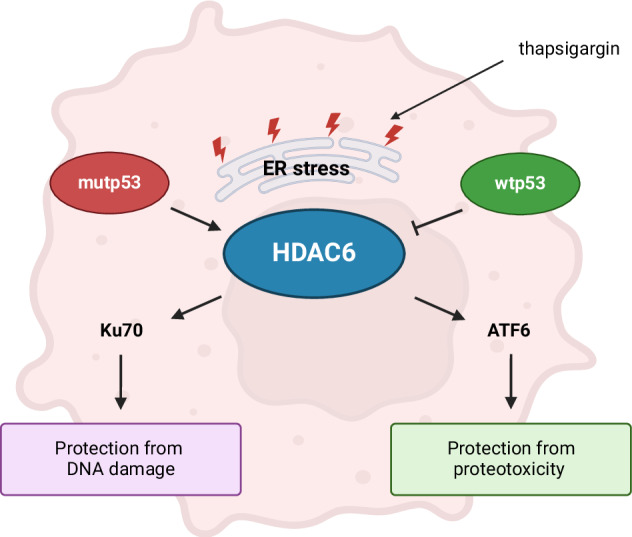

## Introduction

Cancer cells activate multiple protective responses to maintain homeostasis, including the unfolded protein response (UPR) and the DNA damage response (DDR) [1]. The UPR is triggered by ER stress, which can be caused by the accumulation of unfolded proteins in the ER and disruption of Ca²^+^ storage, among other factors. The UPR is orchestrated by three distinct and at the same time interconnected sensors: IRE1α, PERK, and ATF6, that activate multiple pro-survival processes, at least until the stress becomes too strong [2]. The DDR helps ensure genome integrity by recognizing DNA lesions and activating several signaling pathways that are ultimately aimed at mediating DNA repair [3]. The most severe form of DNA damage, a double-strand break (DSB), can be repaired by two main pathways: homologous recombination (HR) and non-homologous end joining (NHEJ). HR, involving molecules such as BRCA1 and Rad51, promotes an error-free DNA repair. In contrast, NHEJ, which involves proteins like DNA-PK, 53BP1, and the Ku70/Ku80 heterodimer, is an error-prone repair mechanism [4]. Consequently, cells that rely on the NHEJ pathway may survive genotoxic insults but are prone to accumulating mutations, thus increasing genomic instability.

A critical stress regulator, helping cells adapt to different types of stress, is HDAC6, which, besides histones, deacetylates several non-histone proteins. HDAC6 positively regulates heat shock protein (HSP) response [5], promotes the formation of aggresomes in response to misfolded protein stress [6], and protects cells from DNA damage caused by genotoxic agents [7].

UPR and DDR are interlinked processes, as UPR sensors not only activate mechanisms that alleviate cells from stress but also control the expression of DDR molecules. For instance, the IRE1α sensor, through its RIDD activity, can promote the degradation of mRNAs encoding DDR molecules [1]. IRE1α has also been reported to be selectively activated by genotoxic stress and protect cells from DNA damage [8]. Meanwhile, ATF6 can sustain the expression level of BRCA1 by increasing mTOR activation and HSP90 expression, allowing HR of DNA damage in colon cancer cells carrying wild-type p53 [9].

p53 is a tumor suppressor protein whose activity is fundamentally incompatible with cancer development, which is why it is often mutated or inactivated in cancer cells, particularly in colon cancer, where p53 mutations occur in about 60% of cases [10]. When mutated, particularly within the core binding domain (CBD), p53 loses its role as the “guardian of the genome” and may even acquire oncogenic functions “gain-of-function” (GOF) [11]. Regarding the relationship between UPR and mutant p53 (mutp53), it has been reported that mutp53 supports the activation of ATF6, thereby increasing cancer cells’ resistance to stress [12]. However, as this UPR sensor is crucial for maintaining lysosomal function, its long-time activation allows the degradation of mutp53 through macroautophagy and/or chaperone-mediated autophagy (CMA) [13].

DDR is also controlled by mutp53, as it has been reported to inhibit ATM and DNA-PK pathways in response to low-dose γ-radiation [14] and to stabilize replication forks, promoting cell proliferation despite the presence of genomic abnormalities [15–17].

Given that mutp53 can influence both UPR and DDR and given the interconnection between these responses, it is crucial to elucidate how mutp53 governs such interplay to assist cancer cells to manage stress. Understanding this may allow to overcome the higher resistance that mutp53-carrying cells offer to stress and potentially tip the balance of these responses from survival to cell death. To address this issue, we exposed colon cancer cells carrying wild-type (wtp53) or the hot-spot p53 mutation (R273H), known to correlate with higher aggressiveness [18] and to play critical roles in chemotherapy-induced colorectal CSC [19], to the ER stressor thapsigargin (TG) and monitored the kinetics of UPR activation with the occurrence of DNA damage in both cell types. We also evaluated how wtp53 and mutp53 could influence the expression of DDR molecules belonging to homologous recombination (HR) and non-homologous end joining (NHEJ) pathways during ER stress and the underlying regulatory mechanisms involved. The results obtained suggest that mutp53-carrying cells exposed to TG displayed different acetylation, expression, and intracellular localization of Ku70, which, together with Ku80, forms a dimer playing a key role in NHEJ DNA repair. We demonstrated that in the acetylation of Ku70 was involved HDAC6, which was upregulated by mutp53 during ER stress, while it was downregulated by wtp53. Moreover, we show that HDAC6 played a pivotal role in activating ATF6, a key component of the unfolded protein response (UPR), which has previously been reported to help mutp53-expressing cancer cells cope with ER stress [12].

In conclusion, this study unveils, for the first time, that the differential regulation of HDAC6 represents a crucial event through which mutp53 modulates the interplay between the UPR and DDR during ER stress, highlighting HDAC6 as a key therapeutic target. Indeed, its inhibition may offer a potential strategy to combat the enhanced resistance of mutp53 cancer cells to treatments inducing ER stress.

## Results

### Mutp53 diminishes the pro-apoptotic effects of the UPR and mitigates DNA damage induced by TG in colon cancer cells

**Fig. 1:**
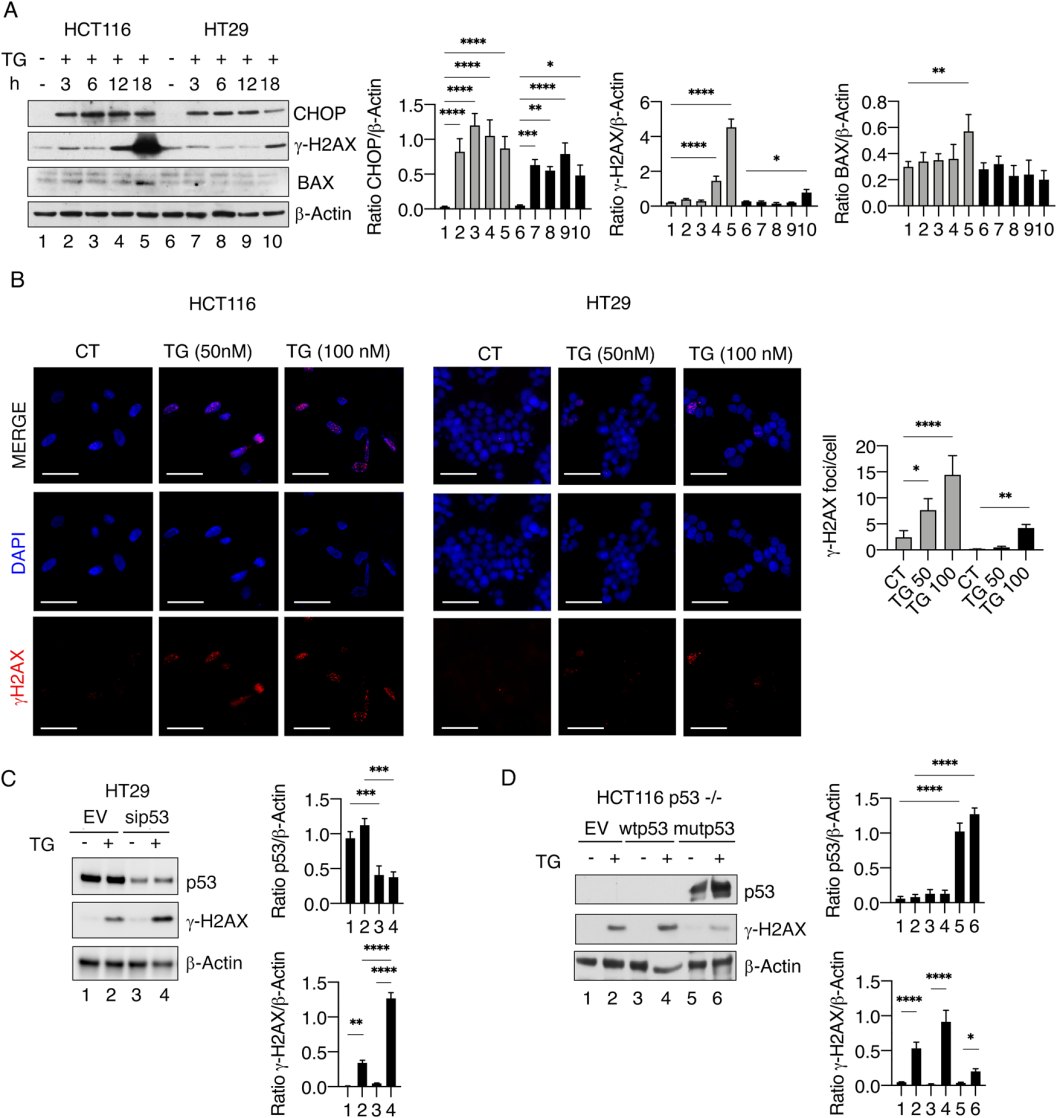
Mutant p53 protects from DNA damage induced by ER stressor TG in colon cancer cell lines. **A** Protein expression levels of CHOP, γ-H2AX, and BAX in HCT116 and HT29 cells treated with TG (100 nM) for the indicated time, as evaluated by western blot analysis. β-Actin was used as a loading control and one representative experiment is shown. The histograms represent the densitometric analysis of the ratio of specific proteins and the appropriate control. The data are represented as the mean plus S.D. from three different experiments. **B** γ-H2AX foci (red) were assessed by IFA in HCT116 and HT29 cells treated with TG for 18 h. DAPI (blue) was used for nuclear staining. One representative experiment out of three is reported. The histograms represent the mean plus S.D. of the number of γ-H2AX foci/cell. Bars = 50 µm. **C** Protein expression levels of mutp53 and γ-H2AX in HT29 cells transfected with pSuper-p53 (sip53) or empty-vector (EV) before treatment with TG. β-Actin was used as a loading control and one representative experiment is shown. The histograms represent the densitometric analysis of the ratio of specific protein/β-Actin. The data are represented as the mean plus S.D. from three different experiments. **D** Protein expression levels of p53 and γ-H2AX in HCT116 p53−/− transfected with pcDNA3-p53 (wtp53), pcDNA3-p53R273H (mutp53) or empty-vector (EV) and then treated or not with TG, as evaluated by western blot analysis. β-Actin was used as a loading control and one representative experiment is shown. Histograms represent the densitometric analysis of the ratio of specific protein/β-Actin. The data are represented as the mean plus S.D. from three different experiments. *p* value *<0.05, **<0.01, ***<0.001, ****<0.0001.

UPR and DDR cooperate to ensure cell survival in the face of stress, as ER stress induces DNA damage and DDR molecules regulate UPR [1]. In this study, we investigated how mutp53 and wtp53 influence the interplay between UPR and DDR in colon cancer cells exposed to ER stress. The kinetic activation of the pro-apoptotic UPR molecules CHOP was evaluated in HCT116 and HT29, colon cancer cells harboring wtp53 and mutp53, respectively, following thapsigargin (TG) treatment and correlated with the induction of DNA damage. As shown in Fig. 1A, by comparing wtp53 and mutp53 cells exposed to TG, we observed a lower increase of CHOP in mutp53 cells HT29 compared to HCT116, particularly after 18 h of treatment, suggesting that mutp53 cells may start recovering at this time. Moreover, we found that the pro-apoptotic molecule BAX was upregulated in wtp53 cells while not in mutp53 cells in which a less intense DNA damage was induced by TG, based on the expression of phosphorylated H2AX (γ-H2AX). To further investigate the DNA damage and the impact of mutp53 on it in stressed cells, the appearance of γ-H2AX-positive foci was investigated by IFA in HT29 and HCT116 as well as in two other colon cancer cell lines harboring mutp53 and wtp53, respectively SW480 and RKO. TG, used at two different doses, induced the formation of a higher number of γH2AX-positive foci in wtp53 HCT116 and RKO compared to HT29 and SW480 mutp53 cells (Fig. 1B and Supplementary Fig. S1), confirming the results obtained by western blot analysis. The role of mutp53 in preventing DNA damage in response to ER stress was then confirmed by experiments in which mutp53 was silenced before exposure to TG (Fig. 1C) or in which mutp53 or wtp53 were overexpressed in HCT116 p53−/− cells undergoing TG treatment (Fig. 1D). Altogether these results suggest that mutp53, besides reducing the pro-apoptotic function of UPR, was able to mitigate DNA damage in colon cancer cells exposed to ER stress. The protective role of mutp53 under stress conditions was further demonstrated by the ability to sustain cell survival when cells were exposed to TG for 18 h, particularly at 100 nM dose, and to prevent apoptotic cell death, based on PARP1 cleavage, pro-caspase 3 reduction and AnnexinV/PI staining (Supplementary Fig. S2A–C).

### Mutp53 sustains the expression level of Ku70/Ku80 and slightly affects that of BRCA1 and Rad51 in colon cancer cells treated with TG

**Fig. 2:**
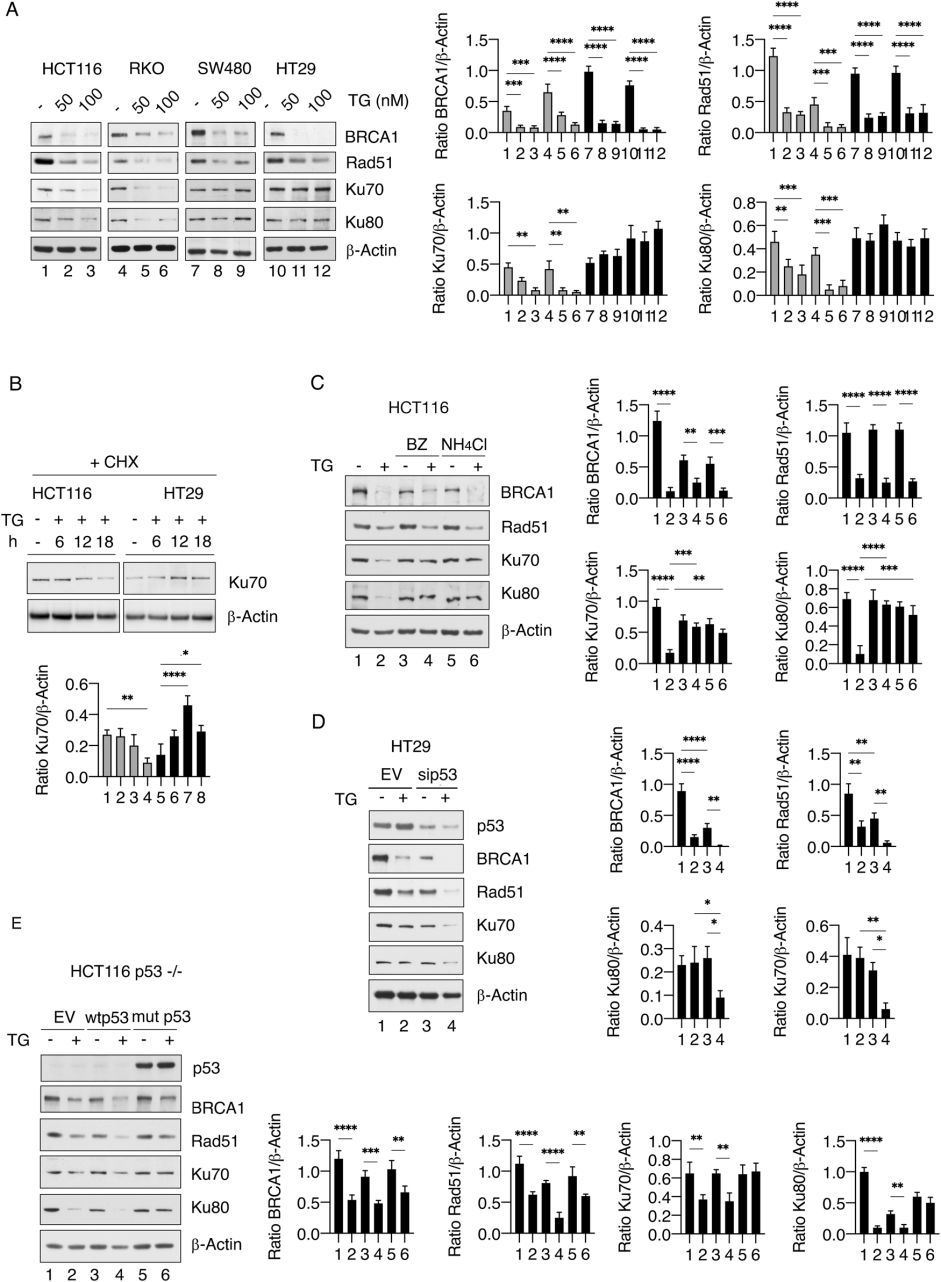
Mutant p53 sustains DNA damage repair protein expression, particularly Ku70 and Ku80, in TG-treated cells. **A** Western blot analysis showing the expression levels of BRCA1, Rad51, Ku70, and Ku80 in HCT116, RKO, SW480, and HT29 cells treated or not with TG. β-Actin was used as a loading control and one representative experiment is shown. The histograms represent the densitometric analysis of the ratio of specific proteins/β-Actin. The data are represented as the mean plus S.D. from three different experiments. **B** Protein expression level of Ku70 in HCT116 and HT29 cells treated with TG (100 nM) for the indicated time in combination with Cycloheximide (CHX). β-Actin was used as loading control and one representative experiment is shown. The histograms represent the densitometric analysis of the ratio of Ku70/β-Actin. The data are represented as the mean plus S.D. from three different experiments. **C** Western blot analysis showing the expression levels of BRCA1, Rad51, Ku70, and Ku80 in HCT116 and HT29 cells treated with TG (100 nM) in combination or not with bortezomib (BZ) or ammonium chloride (NH_4_Cl). β-Actin was used as loading control and one representative experiment is shown. The histograms represent the densitometric analysis of the ratio of specific proteins/β-Actin. The data are represented as the mean plus S.D. from three different experiments. **D** Protein expression levels of mutp53, BRCA1, Rad51, Ku70, and Ku80 in HT29 cells transfected with pSuper-p53 (sip53) or empty-vector (EV) before treatment with TG. β-Actin was used as loading control and one representative experiment is shown. The histograms represent the densitometric analysis of the ratio of specific protein/β-Actin. The data are represented as the mean plus S.D. from three different experiments. **E** Protein expression levels of p53, BRCA1, Rad51, Ku70, and Ku80 in HCT116 p53−/− transfected with pcDNA3-p53 (wtp53), pcDNA3-p53R273H (mutp53) or empty-vector (EV) and then treated or not with TG, as evaluated by western blot analysis. β-Actin was used as loading control and one representative experiment is shown. Histograms represent the densitometric analysis of the ratio of specific proteins/β-Actin. The data are represented as the mean plus S.D. from three different. *p* value *<0.05, **<0.01, ***<0.001, ****<0.0001.

The expression of molecules belonging to HR and/or NHEJ double-strand brake DNA repair was then investigated in mutp53 and wtp53 cells treated with TG. We found that HR molecules such as BRCA1 and Rad51 were reduced after 18 h of TG treatment in both wtp53 and mutp53 cell lines, either at protein (Fig. 2A) and mRNA level (Fig. S2), while Ku70 and Ku80, which form a dimer, were downregulated at protein level in wtp53 cells and slightly upregulated or stable in cells carrying mutp53 (Fig. 2A), with little differences in terms of mRNA expression (Fig. S3). Therefore, we hypothesize that these proteins could have a different stability and may be influenced by different post-translational modifications (PTMs). To address this issue, we compared Ku70 protein stability in wtp53 and mutp53-carrying cells treated with TG by performing a time-course experiment in the presence of cycloheximide (CHX). We found that Ku70 expression level started to decrease following 12 h and was reduced after 18 h of TG treatment in wtp53 cells, while its stability increased after 12/18 h in mutp53 cells (Fig. 2B). Next, we found that both proteasome and lysosomes contributed to Ku70 as well as Ku80 degradation in wtp53 cancer cells treated with TG (Fig. 2C). BRCA1 was also partially degraded by proteasome, while Rad51 was not affected either by proteasome or lysosome inhibition (Fig. 2C). Similar results in the regulation of Ku70 and Ku80 by mutp53 and wtp53 were observed in experiments in which mutp53 was silenced in HT29 cells (Fig. 2D) or wtp53 or mutp53 overexpressed in HCT116 p53−/− cells, before exposure to TG (Fig. 2E). BRCA1 and Rad51 expression levels were also influenced by mutp53 silencing and partially by mutp53 overexpression in these colon cancer cells treated by TG.

### HDAC6 contributes to maintaining high Ku70 expression by promoting its deacetylation in mutp53-carrying cells exposed to TG

**Fig. 3:**
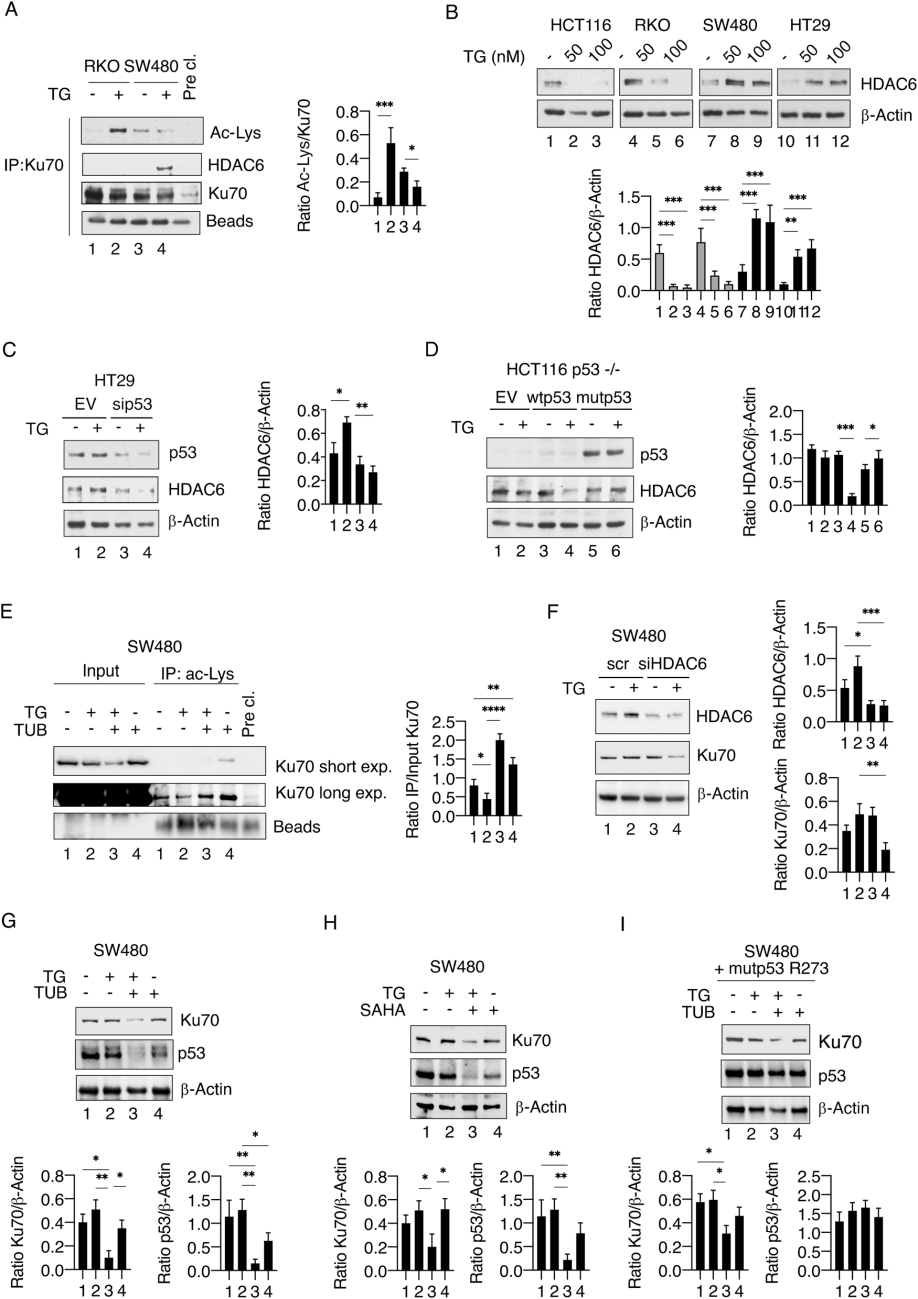
HDAC6 regulation contributes to the different Ku70 acetylation and expression in wild-type and mutant p53 cells treated with TG. **A** Acetylation levels of Ku70 and HDAC6 interaction as evaluated by western blot analysis after immunoprecipitation (IP) with anti-Ku70 antibody in RKO and SW480 cells treated or not with TG (100 nM) for 18 h. Pre-clearing supernatant (Pre-cl.) was used as non-specific binding control. One representative experiment out of three is shown. Histograms represent the mean plus S.D. of the densitometric analysis of the ratio of acetylate-Lysine/Ku70. **B** Western blot analysis showing the expression levels of HDAC6 in HCT116, RKO, and SW480 and HT29 cells treated or not with TG. β-Actin was used as loading control and one representative experiment is shown. The histograms represent the densitometric analysis of the ratio of HDAC6/β-Actin. The data are represented as the mean plus S.D. from three different experiments. **C** Protein expression levels of mutp53 and HDAC6 in HT29 cells transfected with pSuper-p53 (sip53) or empty-vector (EV) before treatment with TG (100 nM). β-Actin was used as loading control and one representative experiment is shown. The histograms represent the densitometric analysis of the ratio of HDAC6/β-Actin. The data are represented as the mean plus S.D. from three different experiments. **D** Protein expression levels of p53 and HDAC6 in HCT116 p53−/− transfected with pcDNA3-p53 (wtp53), pcDNA3-p53R273H (mutp53) or empty-vector (EV) and then treated or not with TG (100 nM), as evaluated by western blot analysis. β-Actin was used as loading control and one representative experiment is shown. Histograms represent the densitometric analysis of the ratio of HDAC6/β-Actin. The data are represented as the mean plus S.D. from three different experiments. **E** Acetylation levels of Ku70 as evaluated by western blot analysis after immunoprecipitation with anti-acetyl-Lysine (ac-Lys) antibody or crude lysate (Input) in SW480 cells pre-treated with tubacin (TUB) and then treated or not with TG for 18 h. Pre-clearing supernatant (Pre-cl.) was used as a non-specific binding control. One representative experiment out of three is shown. Histograms represent the mean plus S.D. of the densitometric analysis of the Ku70 ratio after immunoprecipitation relative to the Input. **F** Protein expression levels of HDAC6 and Ku70 in SW480 cells transfected with HDAC6 siRNA (siHDAC6) or control siRNA-A (scr) before treatment with TG. β-Actin was used as loading control and one representative experiment is shown. The histograms represent the densitometric analysis of the ratio of specific proteins/β-Actin. The data are represented as the mean plus S.D. from three different experiments. **G**, **H** Protein expression levels of Ku70 and mutp53 in SW480 cells exposed or not with TG after pre-treatment with TUB or SAHA, as evaluated by western blot analysis. β-Actin was used as loading control and one representative experiment is shown. The histograms represent the densitometric analysis of the ratio of specific proteins/β-Actin. The data are represented as the mean plus S.D. from three different experiments. **I** Western blot analysis of Ku70 and mutp53 in SW480 transfected with pcDNA3-p53R273H (mutp53) vector, pre-treated with TUB and then treated or not with TG. The histograms represent the densitometric analysis of the ratio of specific protein/β-Actin. The data are represented as the mean plus S.D. from three different experiments. *p* value *<0.05, **<0.01, ***<0.001.

We then evaluated if different PTMs could underly the different stability of Ku70 observed in wtp53 and mutp53-carrying cells undergoing TG treatment. As acetylation has been reported to play a key role in the regulation of Ku70 [20], we evaluated it in wtp53 and mutp53 cancer cells exposed to TG and found that Ku70 was hyperacetylated in wtp53 cells treated by TG compared to the control while deacetylated in cells carrying mutp53 undergoing the same treatment (Fig. 3A).

We then focused on HDAC6, a histone deacetylase playing a key role in protecting cells in response to stress [5], as it has been reported to localize in the cytoplasm and deacetylate several non-histone proteins, including Ku70, influencing its interaction with Bax [21]. As shown in Fig. 3A, HDAC6 interaction with Ku70 increased in mutp53-carrying cells treated with TG and this HDAC was upregulated in mutp53 cells while downregulated in wtp53 cells undergoing this treatment (Fig. 3B). Similarly, HDAC6 was reduced by TG in cells in which mutp53 was silenced (Fig. 3C), or wtp53 overexpressed, while increased in cells overexpressing mutp53 (Fig. 3D). The role of HDAC6 in reducing Ku70 acetylation in mutp53 cells was demonstrated by using the HDAC6 inhibitor tubacin (Fig. 3E) and observing that this treatment prevented Ku70 deacetylation. The silencing of HDAC6 (Fig. 3F), the treatment with tubacin (Fig. 3G) as well as with the HDAC inhibitor SAHA (Fig. 3H), known to also target HDAC6, induced the degradation of Ku70 in TG-treated mutp53 SW480 cells, confirming the correlation between HDAC6 inhibition and Ku70 degradation. To exclude that Ku70 degradation induced by tubacin/TG was due to the downregulation of mutp53 induced by this combination (Fig. 3G, H), we transfected mutp53 in SW480 and found that Ku70 was still reduced after tubacin/TG treatment (Fig. 3I), confirming the role of HDAC6-deacetylation in sustaining its expression. We then repeated some key experiments by using another ER stressor, tunicamycin (TN), and found that mutp53 cells (HT29) displayed a reduced expression of CHOP and γH2AX also in the presence of this ER stressor compared to wtp53 cells (HCT116) (Fig. S4A), and that this occurred in correlation with the expression of HDAC6 (Fig. S4B). Moreover, as for TG treatment, the use of tubacin reduced the expression level of Ku70 and increased γH2AX in TN-treated HT29 cells (Fig. S4C).

### The acetylation of Ku70 promotes its nuclear localization and interactions with key proteins such as importin/exportin and MDM2

**Fig. 4:**
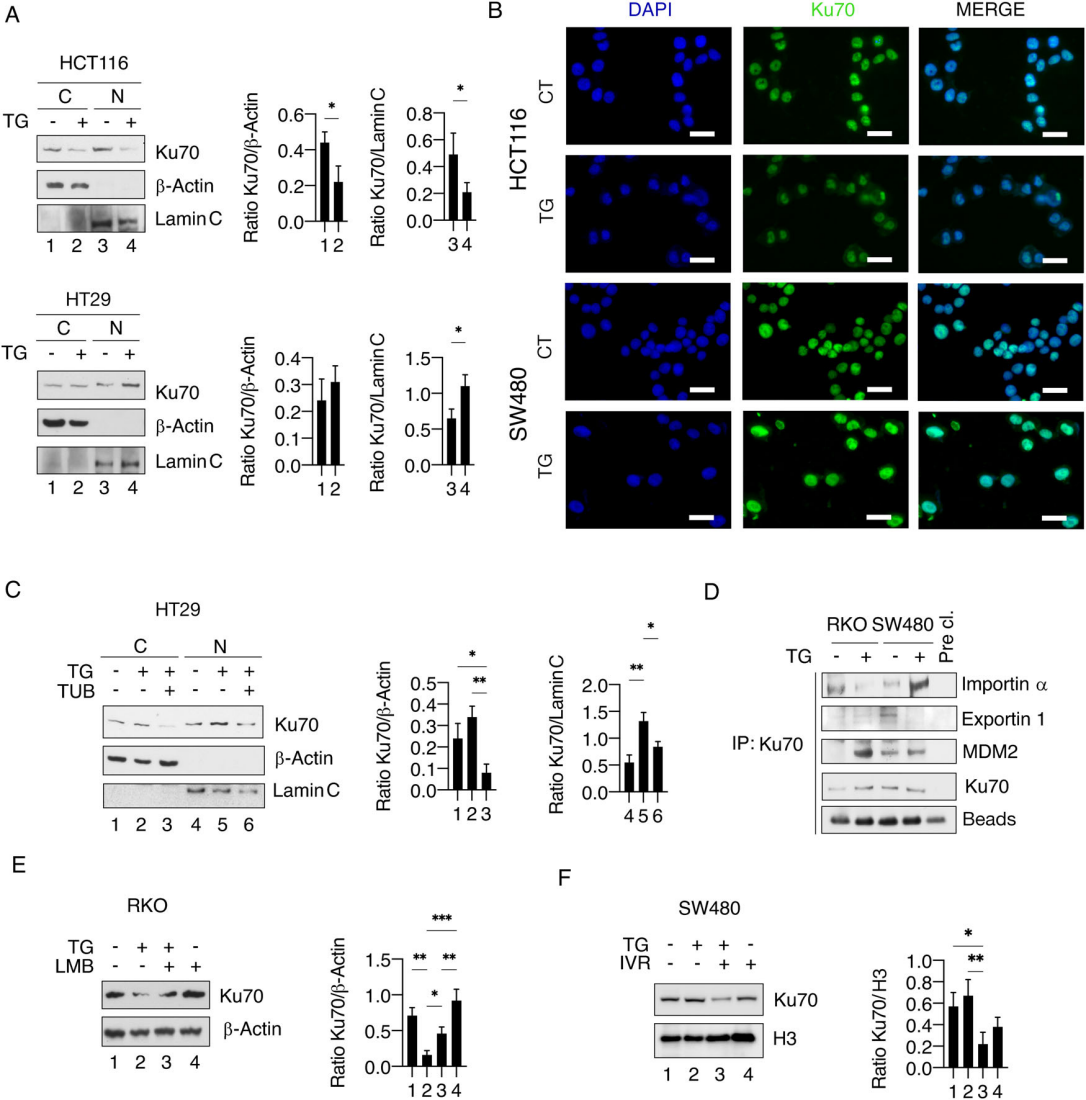
TG influences Ku70 import/export affecting its expression level in wild-type and mutant p53 cells. **A** Ku70 localization was evaluated by western blot analysis performed after nuclear/cytoplasmic fractionation in HCT116 and HT29 cells treated or not with TG (100 nM) for 18 h. β-Actin or Lamin C was used as a loading control and one representative experiment is shown. The histograms represent the densitometric analysis of the ratio of Ku70 and the appropriate control. The data are represented as the mean plus S.D. from three different experiments. **B** Immunofluorescence for Ku70 (green) in HCT116 and SW480 cells treated or not with TG (100 nM). Blue: DAPI staining. Scale bars = 20 µm. **C** Ku70 localization was evaluated by western blot analysis performed after nuclear/cytoplasmic fractionation in HT29 cells pre-treated with tubacin (TUB) before adding TG (100 nM). β-Actin or Lamin C was used as loading control and one representative experiment is shown. The histograms represent the densitometric analysis of the ratio of Ku70 and the appropriate control. The data are represented as the mean plus S.D. from three different experiments. **D** Protein interaction of Importin α, Exportin 1, and MDM2 with Ku70 as evaluated by western blot analysis after immunoprecipitation (IP) in RKO and SW480 cells treated or not with TG (100 nM) for 18 h. Pre-clearing supernatant (Pre-cl.) was used as non-specific binding control. One representative experiment out of three is shown. **E**, **F** Protein expression levels of Ku70 in RKO or SW480 cells treated or not with TG (100 nM) after pre-treatment with leptomycin B (LMB) or ivermectin (IVM) as evaluated by western blot analysis. β-Actin was used as loading control and one representative experiment is shown. The histograms represent the densitometric analysis of the ratio of Ku70/β-Actin. The data are represented as the mean plus S.D. from three different experiments. *p* value *<0.05, **<0.01.

Ku70 acetylation has been reported to affect its nuclear import/export, as in the acetylated status it may less efficiently interact with Importin-α [22]. Accordingly, here we found that TG promoted Ku70 localization in the nuclei in HT29 mutp53-carrying cells, while its nuclear localization decreased in HCT116 wtp53 cells treated with TG (Fig. 4A). These results were confirmed by IFA experiments in which wtp53 and mutp53 cells were analyzed for Ku70 nuclear/cytoplasm localization (Fig. 4B). To confirm that deacetylation and nuclear localization were interconnected effects, we pre-treated HT29 mutp53 cells with tubacin before exposing to TG and found that its nuclear localization was reduced (Fig. 4C).

Consistent with the nuclear localization, we next observed that Ku70 interaction with Importin-α increased in mutp53 cells while decreased in wtp53 cells following TG treatment. On the other hand, the interaction of Ku70 with Exportin 1 was reduced in TG-treated mutp53 cells while not affected in wtp53 cells (Fig. 4D). Ubiquitination and degradation of proteins may be influenced by intracellular localization, as reported for example for proteins such as p21Cip1, whose ubiquitin-mediated degradation induced by ROS could be counteracted by blocking the nuclear export [23] and for wtp53 that when bound to MDM2 resulted in its nuclear export and degradation [24, 25]. In line with these evidences, we found that Ku70 interacted more strongly with the ubiquitin ligase MDM2 in wtp53 cells (Fig. 4D) where its expression was reduced by TG. Accordingly, the treatment of wtp53 cells with the Exportin 1 inhibitor leptomycin B (LMB) partially restored Ku70 expression level in these cells undergoing TG treatment (Fig. 4E) and conversely, the inhibition of Importin-α by ivermectin (IVR) downregulated Ku70 in mutp53 cells TG-treated (Fig. 4F). Altogether these findings suggest that the different acetylation of Ku70 observed in wtp53 and mutp53 cells influenced its interaction with importin/exportin as well as with MDM2, regulating its localization and degradation in colon cancer cells undergoing TG treatment.

### HDAC6 sustains the activation of ATF6 in mutp53-carrying cells treated with TG

**Fig. 5:**
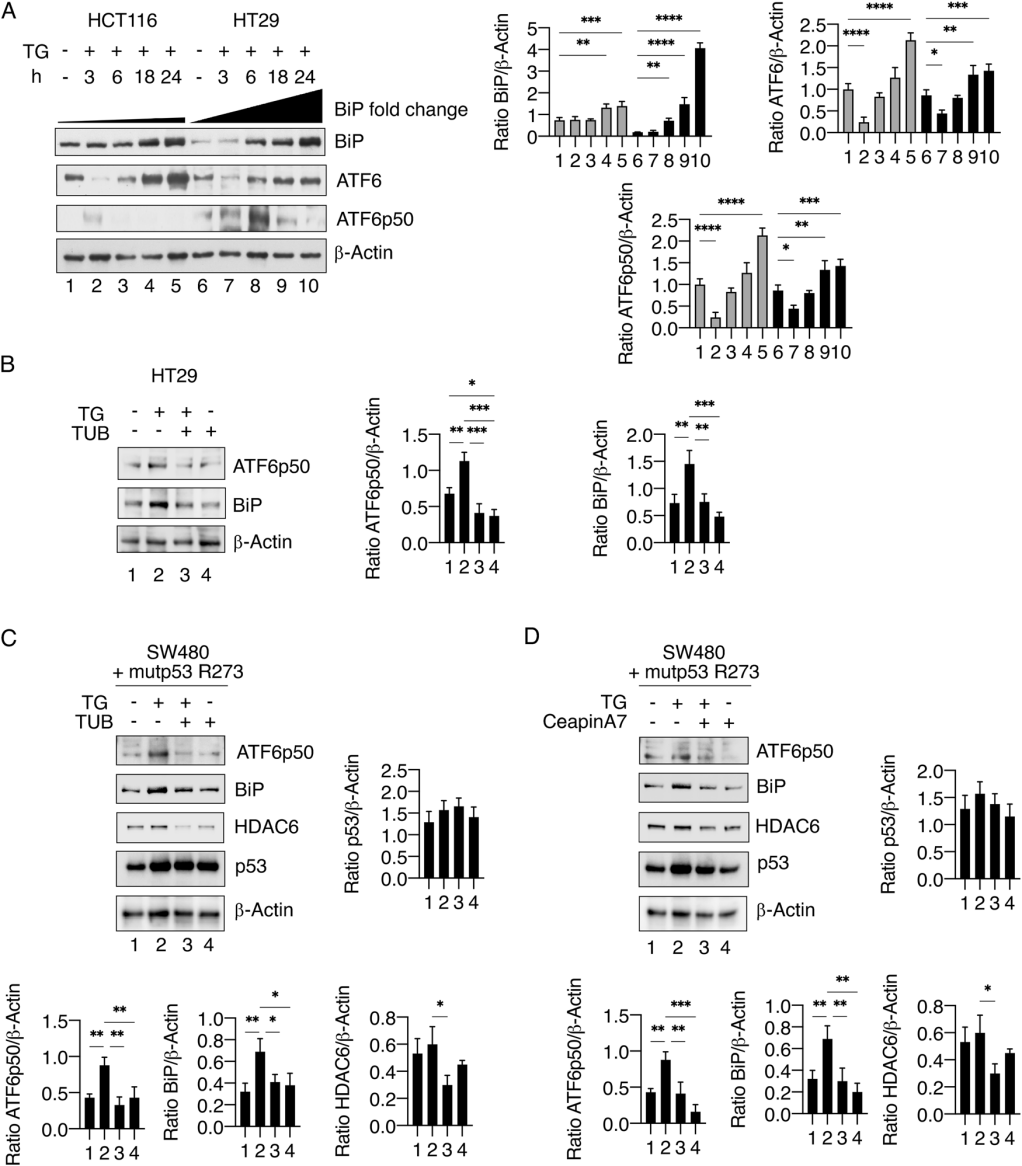
HDAC6 protects from ER stress by sustaining ATF6 activation. **A** Protein expression level of BiP, ATF6, and ATF6p50 in HCT116 and HT29 cells treated with TG (100 nM) for the indicated time as evaluated by western blot analysis. β-Actin was used as loading control and one representative experiment is shown. The histograms represent the densitometric analysis of the ratio of specific protein and the appropriate control. The data are represented as the mean plus S.D. from three different experiments. **B** Protein expression level of ATF6p50 and BiP in HT29 cells pre-treated with tubacin (TUB) and then treated with TG (100 nM) for 18 h. β-Actin was used as loading control and one representative experiment is shown. The histograms represent the densitometric analysis of the ratio of specific protein and the appropriate control. The data are represented as the mean plus S.D. from three different experiments. **C** Western blot analysis of HDAC6, ATF6, BiP, and mutp53 in SW480 transfected with pcDNA3-p53R273H (mutp53) vector and then treated or not with TG after pre-treatment with TUB. β-Actin was used as loading control and one representative experiment is shown. The histograms represent the densitometric analysis of the ratio of specific protein and the appropriate control. The data are represented as the mean plus S.D. from three different experiments. **D** Western blot analysis of HDAC6, ATF6, BiP, and mutp53 in SW480 transfected with pcDNA3-p53R273H (mutp53) vector, then pre-treated with ceapinA7 and treated or not with TG. β-Actin was used as loading control and one representative experiment is shown. The histograms represent the densitometric analysis of the ratio of specific protein and the appropriate control. The data are represented as the mean plus S.D. from three different experiments. *p* value *<0.05, **<0.01, ***<0.001, ****<0.0001.

As mutp53 cells are able to enhance the activation of ATF6 to better resist ER stress [12], and as we have demonstrated here that mutp53 upregulates HDAC6 in stressed cells, we explored the possible interconnection between HDAC6 and ATF6 in mutp53 cells exposed to TG. We first demonstrated that ATF6 was more activated in HT29 mutp53 cells compared to HCT116 wtp53 cells at different time points after TG treatment (Fig. 5A), and that, accordingly, BiP resulted more strongly upregulated in these cells compared to those with wtp53 (Fig. 5A). Then, we evaluated if the stronger activation of ATF6 could be influenced by the HDAC6 and found that the use of the HDAC6 inhibitor tubacin prevented the activation of ATF6p50 and BiP in TG-treated mutp53 cells (Fig. 5B). Given that the tubacin/TG or SAHA/TG combination not only inhibits HDAC6 but also downregulates mutp53 (Fig. 3F, G), we overexpressed mutp53 in HT29 cells prior to treating them with the TG/tubacin combination. This approach allowed us to isolate the effect of HDAC6 on ATF6p50 and BiP, independent of mutp53 downregulation. As shown in Fig. 5C, ATF6p50 and BiP expression levels were still reduced in mutp53-expressing cells treated with the tubacin/TG combination compared to TG alone, supporting HDAC6’s role in sustaining ATF6p50 and BiP. The other way around was also explored, evaluating if ATF6 activation could influence HDAC6 expression. We found that the inhibition of ATF6 by ceapinA7 slightly reduced HDAC6 expression in TG-treated cells (Fig. 5D). Altogether, these results suggest that HDAC6, in addition to prevent DNA damage by sustaining Ku70 expression, contributes to protect cells from ER stress by supporting the activation of ATF6, thus playing a central role in the UPR/DDR interplay. Meanwhile, ATF6 activity only slightly affects HDAC6 expression levels.

## Discussion

Several molecules serve as critical links between the UPR and the DDR, two interconnected adaptive responses that play a central role in regulating cell survival [1]. These pathways enable cancer cells to cope with stress, particularly ER stress and genotoxic stress. Key molecules such as IRE1α and ATF6 within the UPR can influence DDR, allowing cells to coordinate stress responses and promote survival under adverse conditions. Understanding the molecular mechanisms bridging UPR and DDR and their roles offer potential therapeutic targets for disrupting the protective adaptations of cancer cells. Our findings reveal that HDAC6 plays a crucial role in UPR/DDR cross-talk in mutp53-expressing cancer cells exposed to ER stress as it sustained the expression level and the deacetylation of Ku70, facilitating its nuclear localization and preventing its degradation. HDAC6 is known to be the most active histone deacetylase involved in deacetylating non-histone proteins [26]. It has been shown to shuttle between the nucleus and cytoplasm, exerting its effects in both cellular compartments. Importantly, HDAC6 has been reported to mitigate DNA damage also induced by genotoxic agents [7] and sustain the function of mismatch repair proteins, such as MutL homolog 1 (MLH1), through its deacetylating activity [27]. This underscores the significance of HDAC6 in maintaining DNA repair mechanisms and highlights its potential as a therapeutic target for enhancing cancer cells’ sensitivity to genotoxic stress. The modulation of Ku70 stability by HDAC6 is crucial, as Ku70, which dimerizes with Ku80, forms a complex essential for NHEJ, an error-prone DNA repair pathway. While the activation of NHEJ promotes cell survival under stress conditions, it can also contribute to the accumulation of DNA mutations and increase genome instability, highlighting a dual-edged role in cancer progression. Previous studies have reported that γ-irradiation promotes mutp53 stabilization, allowing cell cycle progression in the presence of inefficiently repaired DNA, and that, in mutp53 heterozygous tumors, mutp53 activated the NHEJ pathway to repair DNA damage induced by exposure to low-dose γ-irradiation in vivo, thereby promoting genomic instability [14, 28].

Here, we show that, differently from mutp53 cells, in wtp53-expressing cells, Ku70 underwent degradation during ER stress, through both proteasomal and lysosomal routes, potentially involving Chaperone-Mediated Autophagy (CMA), as TG has been reported to reduce autophagy [29]. Although the KFERQ motif, a recognition sequence for CMA, is not present in Ku70, its acetylation may induce conformational changes that render it targetable by CMA. This possibility represents an intriguing avenue for further investigation, as it may reveal additional regulatory mechanisms governing Ku70 stability in the context of cellular stress responses.

**Fig. 6:**
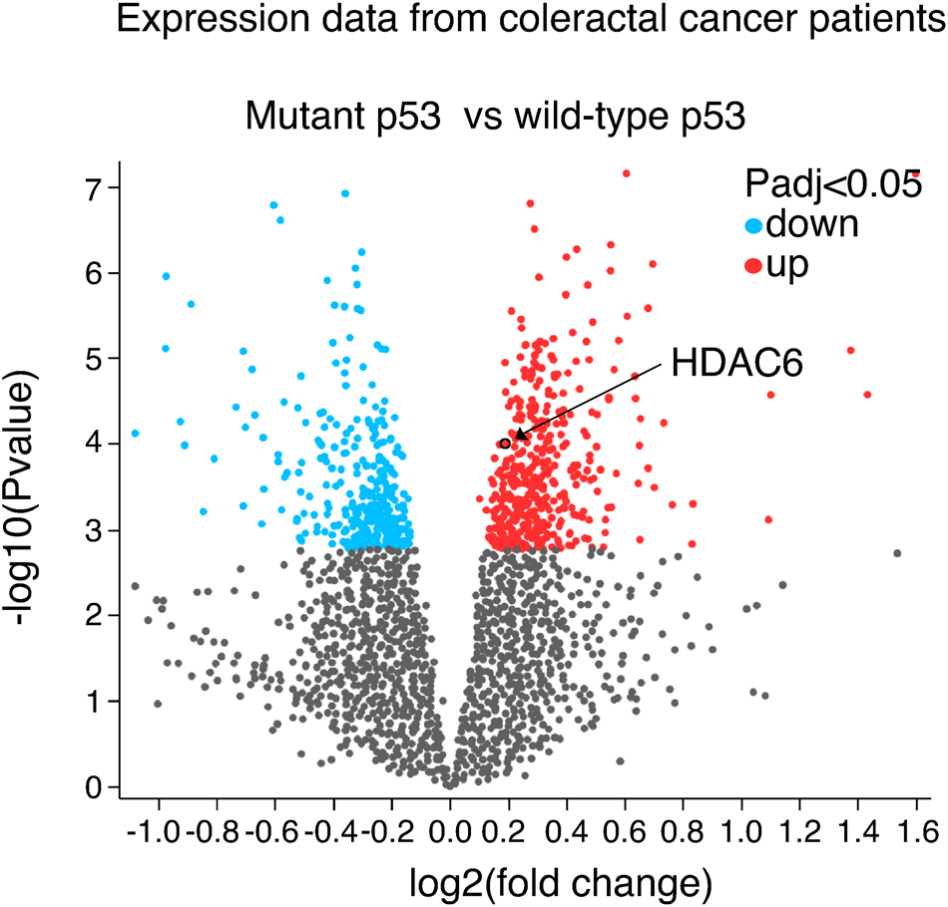
HDAC6 is upregulated in colorectal cancer patient samples with mutated p53 compared to those with wild-type p53. Volcano plot of differentially expressed genes between mutant p53 and wild-type p53 primary tumor samples from colorectal cancer patients, obtained from GEO2R analysis of an expression array dataset. Each point in the plot represents a gene, with the x-axis showing the magnitude of change (log2 fold change) and the y-axis indicating statistical significance (−log10 *P* value). Highlighted genes are significantly differentially expressed at an adjusted *p* value cutoff of 0.05 (red = upregulated, blue = downregulated). The point representing HDAC6 is marked by an arrow (log2 fold change = 0.189, −log10 *P* value = 4).

As another protective mechanism, HDAC6 has been shown to activate HSF1, resulting in the upregulation of heat shock proteins (HSPs) in response to ubiquitinated cellular aggregates [30]. In the present study, we also demonstrate that HDAC6 upregulation in mutp53 cells exposed to TG contributes to the activation of the UPR sensor ATF6, which aids mutp53 cancer cells in managing ER stress. All these findings underscore the pivotal role of HDAC6 in modulating several critical cellular responses to stress, emphasizing its function as a key regulator of cellular adaptation. Targeting HDAC6 might thus represent a promising therapeutic strategy to sensitize mutp53 cancer cells to ER stress and diminish their genomic instability, enhancing treatment efficacy and improving outcomes for patients with cancers harboring mutp53. Interestingly, in accordance with our findings, HDAC6 is among the molecules upregulated by mutp53 in primary colon tumors, as shown in the volcano plot represented in Fig. 6 shows.

Sustaining HDAC6 expression seems to be another of the end-less strategies put in place by mutp53 to help cell survival, which also fuels the accumulation of DNA mutations through the activation of the NHEJ pathway. The deacetylation of HSPs may be induced by HDAC6 [31] and this could contribute to Ku70 stabilization in mutp53 cells. It will be important to investigate this aspect in future studies, as Ku70 is an HSP client protein [32], and HSPs, such as HSP90, can establish a positive feedback loop with HDAC6 [33].

Sustaining HSP function in mutp53 cancer cells undergoing ER stress may have numerous other implications, given the great number of pro-survival and oncogenic molecules known to be HSP clients [34].

In conclusion, this study demonstrates that mutp53 limits DNA damage induced by ER stress through maintaining Ku70 expression by upregulating HDAC6. This regulation bolsters cellular resistance to ER stress and contributes to ATF6 activation, revealing an intricate mechanism by which cancer cells with mutp53 enhance survival under stress conditions. Given HDAC6’s pivotal role in DNA repair and cellular stress management, it emerges as a potential therapeutic target to reduce cancer cell survival and limit progression under stressful conditions.

## Materials and methods

### Cell cultures and treatments

Mutant (mut) p53 HT29 (p53R273H), kindly provided by Prof. N. Merendino (Tuscia University, Viterbo Italy), and SW480 (p53R273H and p53P309S), wild-type (wt) p53 RKO and wtp53 and p53−/− HCT116, kind gift from prof. B. Vogelstein (Johns Hopkins University, Baltimore, MD), human colon cancer cell lines were maintained in DMEM (PAN-Biotech, Germany, P04-03600) supplemented with 10% Fetal Bovine Serum (FBS) (Sial, Italy), L-glutamine (100 μg/ml) (Aurogene, Italy, AU-X0550-100), streptomycin and penicillin (100 U/ml) (Aurogene, AU-L0022-100) at 37 °C in a 5% CO_2_ incubator. Cells were always detached using Trypsin-EDTA solution (Aurogene, AU-L0940-100). Cells were plated in 12-well plates at a density of 10^5^ cells/well, and the following day, treated with thapsigargin (TG, MedChemExpress, NJ, USA, HY-13433) for the indicated times and doses (50–100 nM) chosen based on previous experiments [9] or with tunicamycin (3 μg/ml, TN), Sigma-Aldrich, MO, USA T7765. In some experiments, cells were pre-treated for 1 h with SAHA (5 μM, MedChemExpress, HY-10221/CS-0589) or tubacin (TUB, 10 μM, MedChemExpress, HY-13428), pan-HDAC inhibitor and specific HDAC6 inhibitor, respectively. CeapinA7 (18 μM, Sigma-Aldrich, SML233) was used as a 1 h pre-treatment for ATF6 signaling inhibition. To inhibit nuclear import/export, cells were pre-treated for 1 h with ivermectin (IVR, MedChemExpress, HY-15310) or leptomycin B (LMB, Sigma-Aldrich, L2913). After all pretreatments, TG or TN was added to the culture for an additional 18 h without removing the specific inhibitor. Bortezomib (BZ, 10 nM, Sigma-Aldrich, 5.04314) or ammonium chloride (NH_4_Cl) (20 mM, Sigma-Aldrich, A0171), proteasome and lysosome inhibitors, respectively, were used the last 4 h of treatment to assess proteasomal and autophagic activity. DMSO-treated cells were used as control.

### Knockdown by short interfering RNA (siRNA)

HT29 cells were plated on 12-well plates at a density of 5 × 10^4^ cells/well, and the following day, control pSuper and pSuper-p53 (for p53 interference, sip53) [35] vectors were transfected using Lipofectamine 3000 (Thermo Fisher Scientific, MA, USA, L3000001) according to the manufacturer’s instructions. HDAC6 knockdown was performed by specific HDAC6 siRNA (Santa Cruz Biotechnology, TX, USA, sc-35545) transfection using INTERFERin transfection reagent (Polypolus Transfection, Illkirch-Graffenstaden, France) according to the manufacturer’s instructions. Control siRNA-A (Santa Cruz Biotechnology, sc-37007) was used as a scrambled control (scr). The day after transfections, cells were treated with TG for the indicated experiments.

### Transfection and plasmids

HCT116 p53−/− cells were plated on 12-well plates at a density of 5 × 10^4^ cells/well. After 24 h, cells were transfected with 0.5 μg of pcDNA3, pcDNA3-p53R273H, or pcDNA3-p53 [36] vector using Lipofectamine 3000 according to the manufacturer’s instructions. The day after transfection, cells were treated with TG for an additional 18 h.

### Cell viability

The cell viability was evaluated by a Trypan Blue (Sigma-Aldrich, 72571) exclusion assay after treatments. Experiments were performed in triplicate and repeated at least three times.

### Annexin/PI staining

After TG treatments, HCT116 and HT29 cells were washed with ice-cold PBS, resuspended in annexin V binding buffer, and subsequently stained with annexin V–fluorescein isothiocyanate (FITC) and PI (BD Pharmingen, San Jose, CA, USA, 556547), according to the manufacturer’s recommendation. The cells were analyzed by FACSCalibur (BD Biosciences, East Rutherford, NJ, USA). Data are representative of at least three independent experiments and analyzed by CellQuest (BD Biosciences, East Rutherford, NJ, USA). Debris and dead cells were excluded from the analysis, gating live cells in a forward versus side scatter (FSC vs. SSC) density plot. For each analysis, 10,000 events were recorded.

### RNA isolation and quantitative real-time polymerase chain reaction (qRT-PCR)

After treatments, total RNA was isolated with TRIzol™ Reagent (Invitrogen, CA, USA, 15596026) according to the manufacturer’s instructions. Next, reverse transcription was carried out by using the SensiFAST cDNA Synthesis kit (Meridian, OH, USA, BIO-65054) and Real-Time PCR with the SensiFast SYBR Lo-ROX kit (Meridian, BIO-94020) was performed. Primers were purchased from Sigma-Aldrich.

Primer used:

BRCA1 Fw′-5′-GACTGTTTATAGCTGTTGGAAG-3′

BRCA1 Rv-5′-TTTGGAAGTGTTTGCTACC-3′

RAD51 Fw-5′-TTAGTTCCAATGGGTTTCAC-3′

RAD51 Rv-5′-CCACCTTGAAGTAGTTTGTC-3′

KU70 Fw-5′--AAGAAGAGTTGGATGACCAG-3′

KU70 Rv-5′-GTCACTTCTGTATGTGAAGC-3′

KU80 Fw-5′-CAGTGAGAGTCTGAGAAAAC-3′

KU80 Rv-5′-TAGGCTGCAATCCTTATAGAC-3′

B2M Fw-5′-AAGGACTGGTCTTTCTATCTC-3′

B2M Rv-5′-GATCCCACTTAACTATCTTGG-3′

### Indirect immunofluorescence assay

Cells were seeded on coverslips and the next day treated with TG (50 and 100 nM) for 18 h. Then, the cells were washed with phosphate-buffered saline (PBS), incubated with 2% paraformaldehyde (Electron Microscopy Science, PA, USA, 157-8) for 30 min, permeabilized with 0.1% Triton X-100 (Sigma-Aldrich, T8787) for 5 min and, after three washes, incubated with 1% glycine and 3% bovine serum albumin (BSA, PanReac AppliChem, Germany, A1391,0100) for an additional 30 min. Subsequently, the cells were incubated with mouse monoclonal anti-γ-H2AX (phosphor-Ser 139, Santa Cruz Biotechnology, sc-517) and rabbit polyclonal anti-Ku70 (Proteintech, IL, USA, 10723-1-AP) for 1 h at room temperature (RT). After three washes, the coverslips were incubated with polyclonal conjugated-Cy3 sheep anti-mouse antibody (Jackson ImmunoResearch, UK, 515-165-062) and polyclonal fluorescein (FITC)-conjugated goat anti-rabbit (Jackson Immuno Research Labs, 111-095-045) for 30 min at RT, stained with DAPI (Sigma-Aldrich) for 1 min at RT and mounted on slides. Slides were analyzed with an Apotome Axio Observer Z1 inverted microscope (Zeiss, Germany) equipped with an AxioCam MRM Rev.3 at 40× magnification. Foci number analysis was performed by ImageJ software (1.47 version, NIH, Bethesda, MD, USA).

### Nuclear/Cytosolic fractionation

The separation of the nuclear extract from the cytoplasmic fraction was performed using the Nuclear/Cytosol Fractionation Kit (BioVision, CA, USA, K266-25) following the manufacturer’s instructions.

### Western blot analysis

The preparation of whole-cell protein lysates and western blotting analysis were performed as previously described [37]. Briefly, the cells were washed in PBS, lysed in RIPA buffer, and centrifuged at 14,000 rpm for 20 min. The protein concentration was measured by using the Bio-Rad Protein Assay (Bio-Rad, CA, USA, 5000206) and then equal amounts of protein lysates were subjected to electrophoresis on 4%–12% NuPage Bis-Tris gels (Life Technologies, N00322BOX) according to the manufacturer’s instructions. Subsequently, the gels were transferred on nitrocellulose membranes (Amersham, Germany, 10600003) for 1 h in Tris-Glycine buffer. The membranes were blocked in PBS–0.1% Tween20 solution containing 3% of BSA, probed with specific antibodies, and developed using ECL Blotting Substrate (Advansta, K-12045-D20). The following primary antibodies were used: rabbit polyclonal anti-BiP (Proteintech, 11587-1-AP); rabbit polyclonal anti-CHOP (Proteintech, 15204-1-AP); mouse monoclonal anti-γ-H2AX (phospho-Ser 139, Santa Cruz Biotechnology, sc-517); mouse monoclonal anti-BAX (Proteintech, 60227-1-Ig); mouse monoclonal anti-p53 (clone DO-1, Santa Cruz Biotechnology, sc-126); mouse monoclonal anti-PARP1 (Proteintech, 66520-1-Ig); mouse monoclonal anti-caspase 3 (Santa Cruz Biotechnology, sc-56053); rabbit polyclonal anti-BRCA1 (Proteintech, 22362-1-AP); rabbit polyclonal anti-Rad51 (Proteintech, 14961-1-AP); rabbit polyclonal anti-Ku70 (Proteintech, 10723-1-AP); mouse monoclonal anti-Ku80 (B-1, Santa Cruz Biotechnology, sc-5280); rabbit polyclonal anti-HDAC6 (Proteintech, 12834-1-AP); mouse monoclonal anti-Exportin 1 (Proteintech, 66763-1-Ig); rabbit polyclonal anti-Importin α (Proteintech, 10819-1-AP); rabbit polyclonal anti-ATF6 (Proteintech, 24169-1-AP). Mouse monoclonal anti-β-Actin (Sigma-Aldrich, A5316), rabbit polyclonal anti-eIF2α (Cell Signaling Technology, 9722), mouse monoclonal anti-LaminA/C (sc-7292), and rabbit monoclonal anti-Histone H3 (Cell Signaling, 4499) were used as loading control. The goat anti-Mouse IgGP Peroxidase Conjugate (Sigma-Aldrich, 401215) and the goat anti-Rabbit IgG Peroxidase Conjugate (Sigma-Aldrich, DC03L) were used as secondary antibodies. All the primary and secondary antibodies used in this study were diluted in a PBS–0.1% Tween 20 solution containing 3% BSA.

### Immunoprecipitation assay

To perform the immunoprecipitation assay, 5 × 10^6^ RKO and SW480 cells, treated or not with TG for 18 h, were lysed in 500 μl of RIPA buffer and centrifuged at 14,000 rpm for 30 min at 4 °C. Immunoprecipitation was performed using Protein A/G PLUS-Agarose (Santa Cruz biotechnology, sc-2003) according to the manufacturer’s instructions. Briefly, cell lysate pre-clearing was performed by adding 20 μl of Protein A/G PLUS-Agarose to 500 μg of cellular proteins for 1 h overnight at 4 °C on a rocker platform. After centrifugation, supernatants were transferred to a new tube and incubated with 1–3 μl of primary antibody overnight at 4 °C on a rocker platform. Then, samples were incubated with 20 μl of resuspended volume of Protein A/G PLUS for 1 h at 4 °C on a rocker platform. Precipitated proteins were collected after centrifugation, washed three times in lysis buffer, and analyzed by western blot analysis.

### Densitometric analysis

The quantification of protein bands was performed by densitometric analysis using the Image J software (1.47 version, NIH, Bethesda, MD, USA).

### GEO dataset analysis

To identify genes differentially expressed between mutant and wild-type p53 groups, GEO2R was used to analyze samples from the GEO Series GSE41258 [38, 39], which includes expression arrays from biological specimens of patients with colonic neoplasms. Briefly, only primary tumor samples were selected, and groups were defined based on p53 mutation status, with 92 samples in the mut p53 group and 53 samples in the wt p53 group. GEO2R was run with default parameters, and the results were adjusted for multiple testing using a False Discovery Rate (FDR) cutoff of 0.05.

### Statistical analysis

The results are represented as the mean plus standard deviation (S.D.) of at least three independent experiments, and statistical analyses were performed with Graphpad Prism software (Graphpad Software Inc.). The two-tailed Student *t-*test or a nonparametric 1-way analysis of variance (ANOVA) test were used to demonstrate statistical significance. Difference was considered statistically significant when *p* values were at least <0.05.

## Supplementary information

Supplementary Figures

Original blots

## Data Availability

Research data are stored in an institutional repository and will be shared upon reasonable request to the corresponding author.
