# Supplementary material for: Mutant p53 upregulates HDAC6 to resist ER stress and facilitates Ku70 deacetylation, which prevents its degradation and mitigates DNA damage in colon cancer cells
Source: Cell Death Discov. 2025 Apr 10;11:162. doi: 10.1038/s41420-025-02433-9 (PMC11985993; doi:10.1038/s41420-025-02433-9)
Supplement: Supplementary file 1 — Supplementary Figures [file 41420_2025_2433_MOESM1_ESM.pdf]

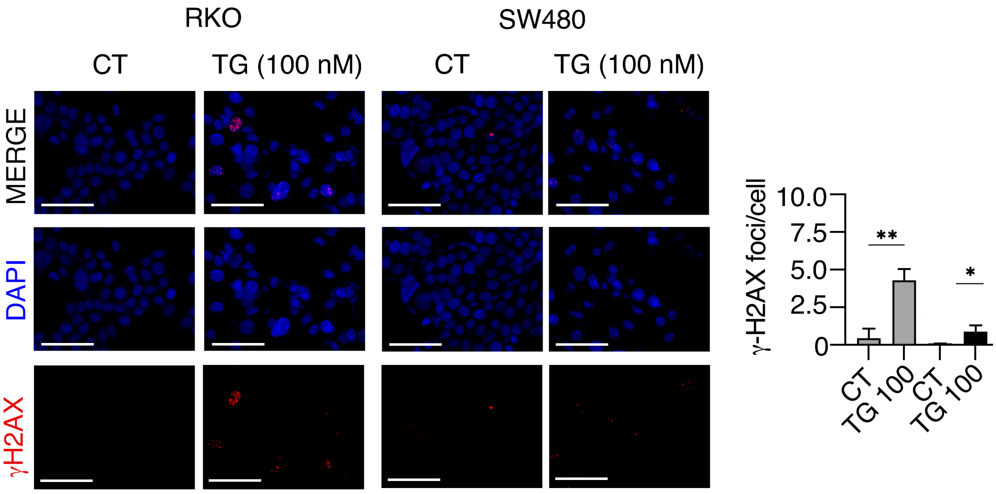

Figure S1

Figure S1. TG induces stronger DNA damage in wtp53 compared to mutp53 colon cancer cells. γ-H2AX foci (red) were assessed by IFA in RKO and SW480 cells treated with TG. DAPI (blue) was used for nuclear staining. One representative experiment out of three is reported. The histograms represent the mean plus S.D. of the number of γ-H2AX foci/cell. Bars = 50 µm. *p* value * <0.05, ** <0.01.

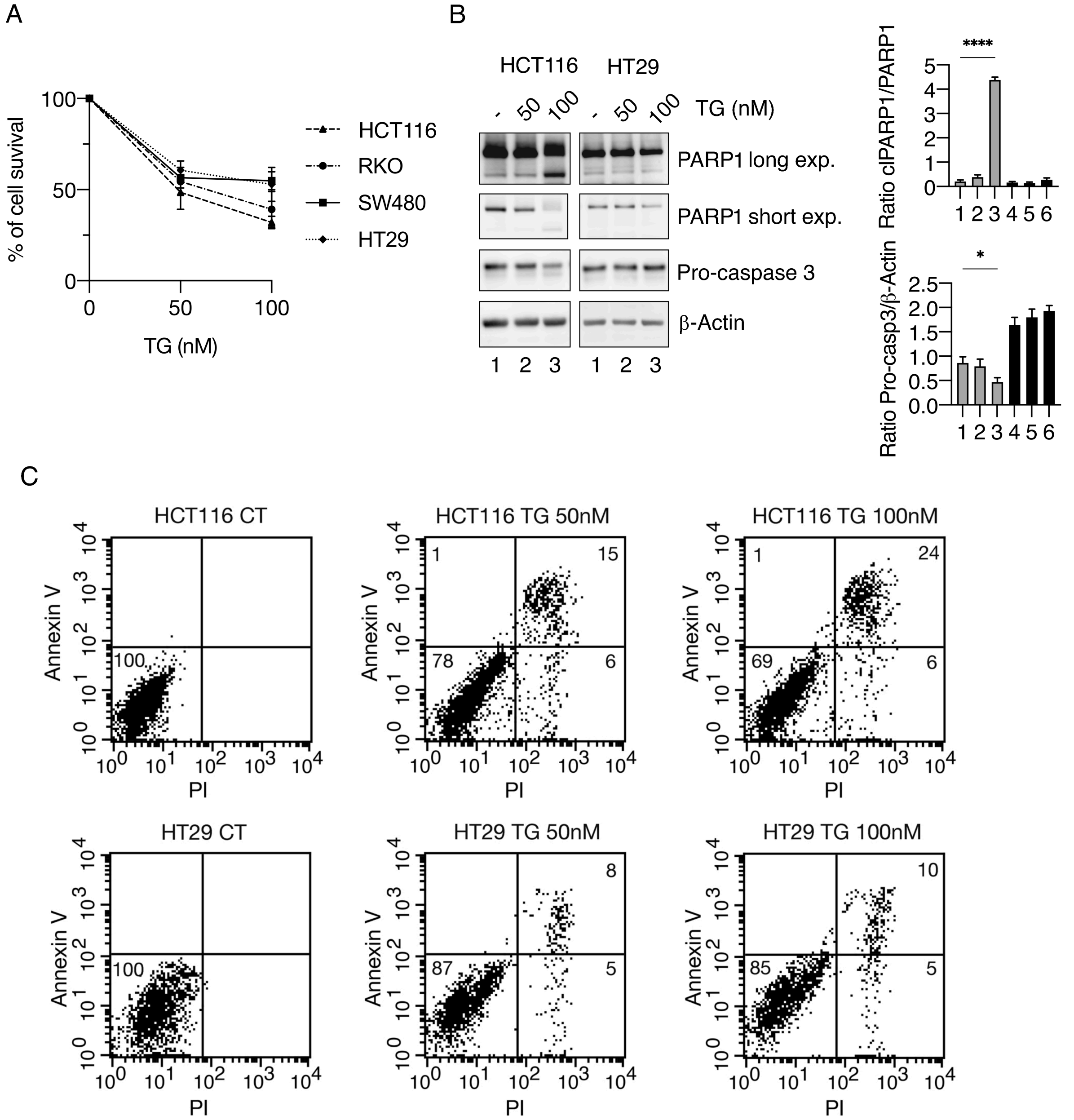

Figure S2

Figure S2. Effects of TG on cell viability of wtp53 and mutp53 colon cancer cells. (A) Mutp53 SW480 and HT29 and wtp53 RKO and HCT116 cell lines were treated with TG (50-100 nM). Cell viability was measured after 18 h of treatment by a Trypan Blue exclusion assay. The points represent the mean ± S.D. of live cells as a percent of untreated control cells from three different experiments. (B) Western blot analysis showing the expression levels of PARP1 and pro-caspase 3 in HCT116 and HT29 cells treated or not with TG. β-Actin was used as a loading control and one

representative experiment is shown. The histograms represent the densitometric analysis of the ratio of specific protein/β-Actin. The data are represented as the mean plus S.D. from three different experiments. (C) HCT116 and HT29 cells treated with TG were double-stained with annexin V/PI and analyzed by FACS analysis. One representative experiment is shown and the mean of the percentage of live (annexin-V-negative, PI-negative), early apoptotic (annexin-V-positive, PI-negative), late apoptotic (annexin-V-positive, PI-positive), and dead cells (annexin-V-negative, PI-positive) is indicated. *p* value * <0.05, **** <0.0001.

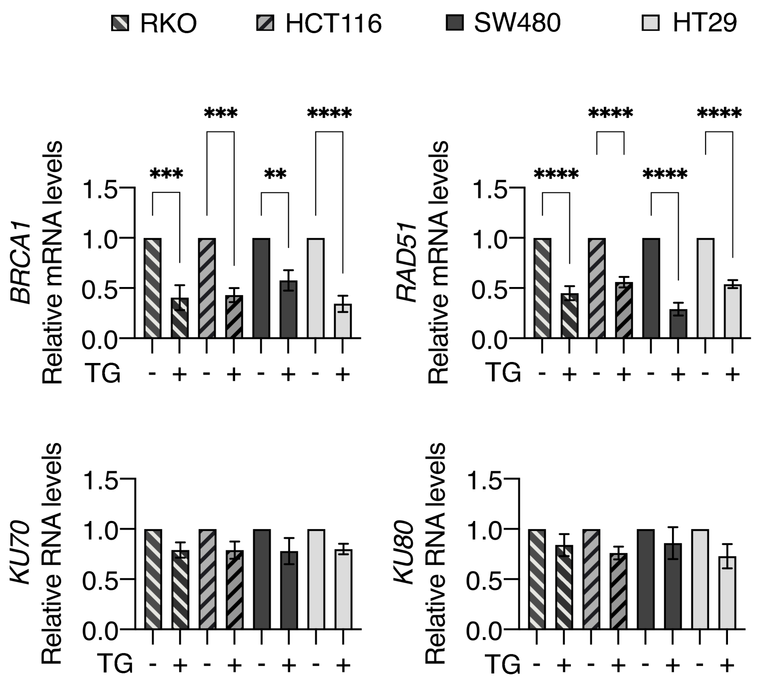

Figure S3

Figure S3. Transcriptional regulation of DDR proteins by TG. qRT-PCR of *BRCA1*, *RAD51, KU70,* and *KU80* in HCT116, RKO, SW480 and HT29 cells treated with TG (100 nM) for 36 h or left untreated as control. Data are expressed relative to the mean of the starting concentration of the reference gene *B2M*. The histograms represent the mRNA expression levels of the genes in three different experiments. Data are represented as the mean relative to the control ± S.D. *p* value ** <0.01, *** <0.001, **** <0.0001.

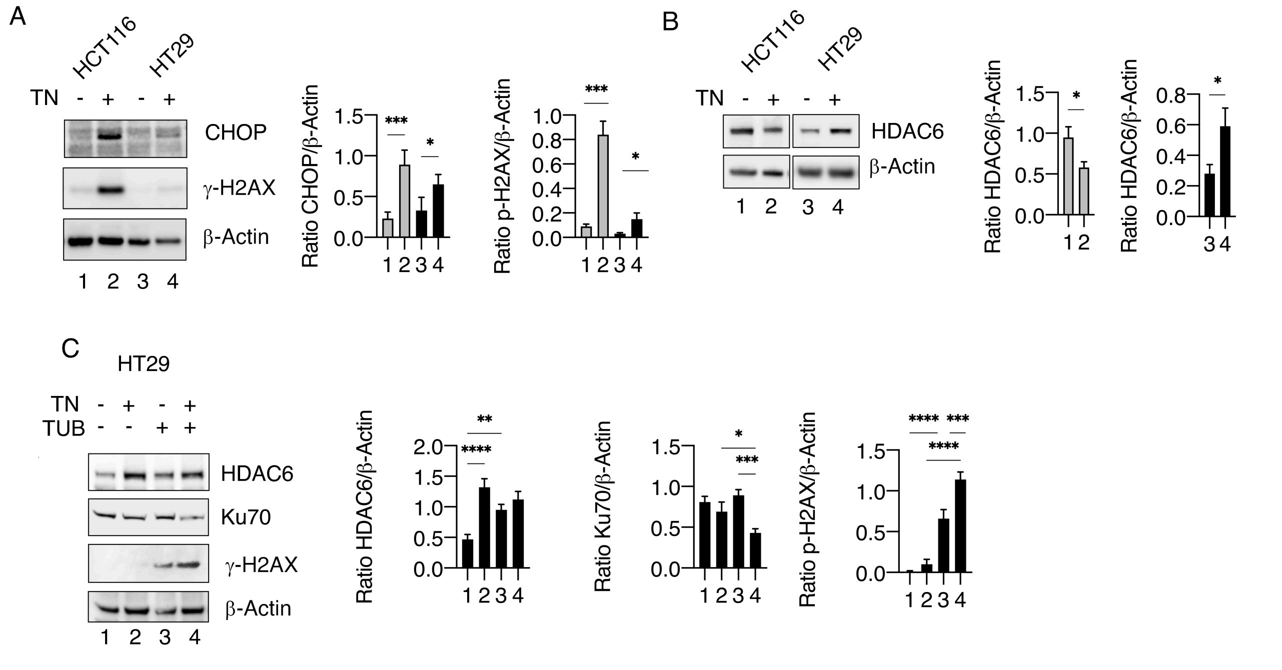

Figure S4. HDAC6 protects from DNA damage and sustains Ku70 expression in mutp53 cells also after tunicamycin-induced ER stress. (A, B) Western blot analysis showing the expression levels of CHOP, γ-H2AX, and HDAC6 in HCT116 and HT29 cells treated or not with TN (3 μg/ml) for 18 h and (C) protein expression levels of HDAC6, Ku70, and γ-H2AX in HT29 cells pre-treated with tubacin (TUB) before treatment with TN. β-Actin was used as a loading control and one representative experiment is shown. The histograms represent the densitometric analysis of the ratio of specific protein/β-Actin. The data are represented as the mean plus S.D. from three different experiments. *p* value * <0.05, ** <0.01, *** <0.001, **** <0.0001.
