# Supplementary material for: Mutant p53 upregulates HDAC6 to resist ER stress and facilitates Ku70 deacetylation, which prevents its degradation and mitigates DNA damage in colon cancer cells
Source: Cell Death Discov. 2025 Apr 10;11:162. doi: 10.1038/s41420-025-02433-9 (PMC11985993; doi:10.1038/s41420-025-02433-9)

Fig.1A

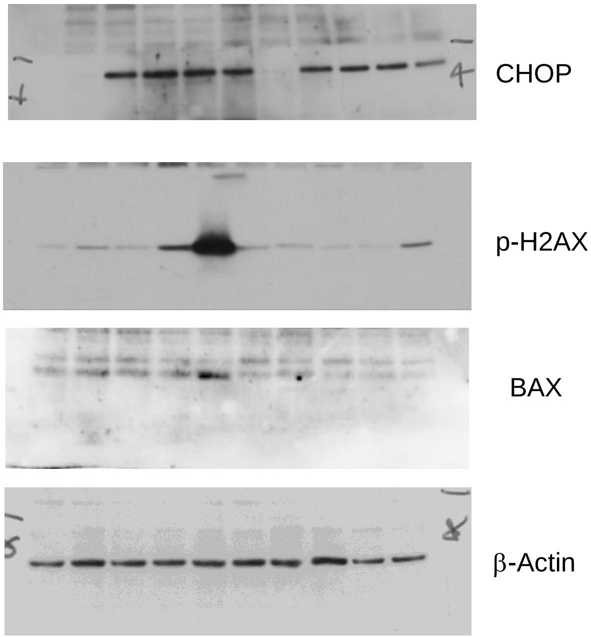

Fig.1C

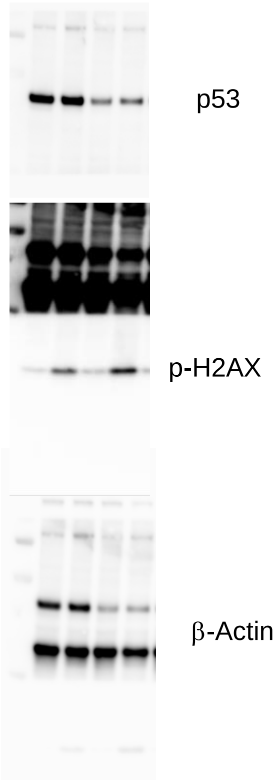

Fig.1D

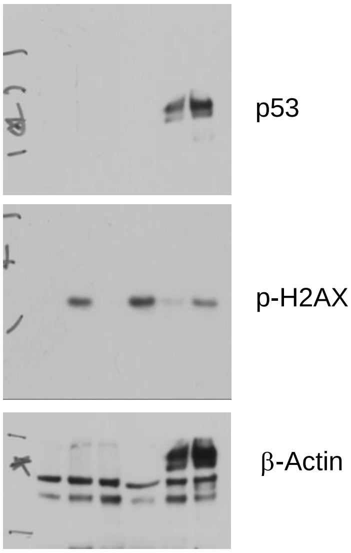

Fig.2A

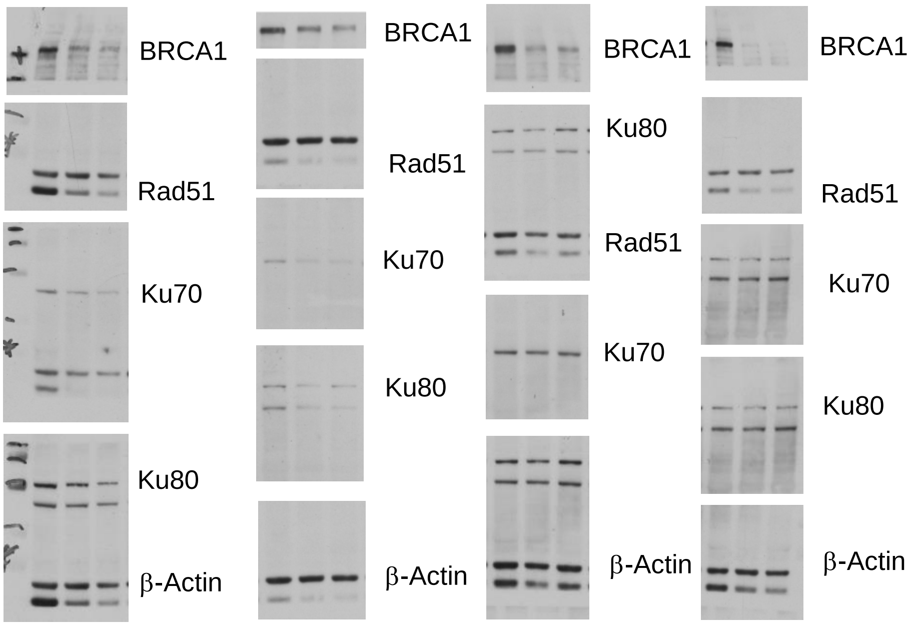

Fig.2B

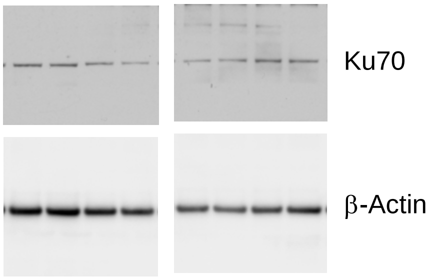

Fig.2C

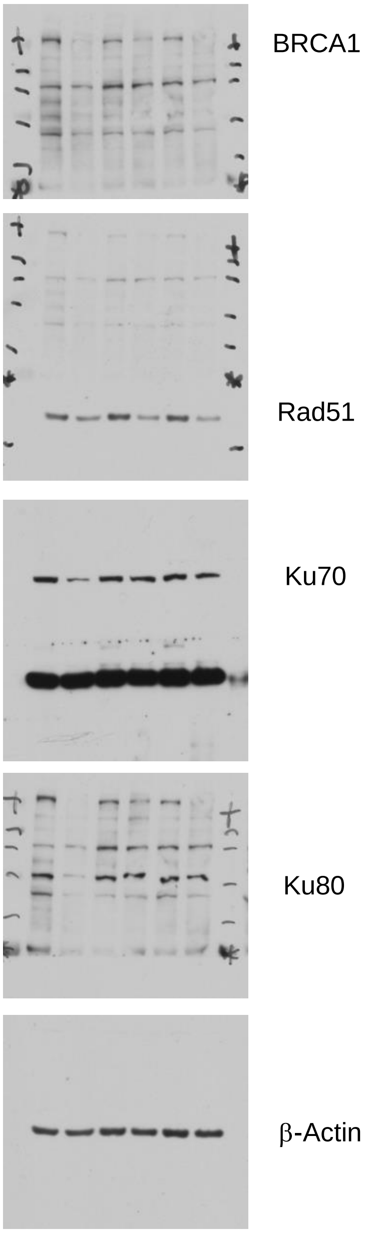

Fig.2D

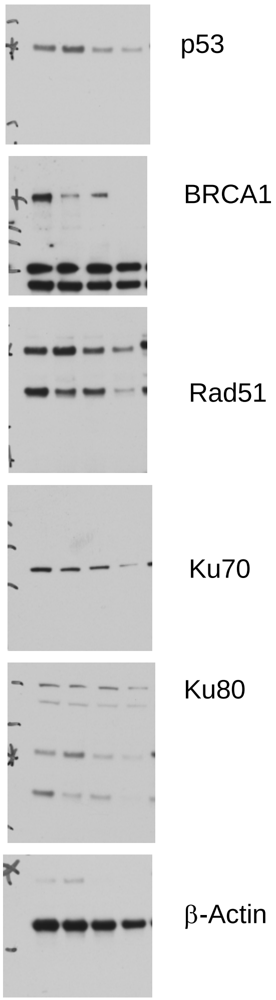

Fig.2E

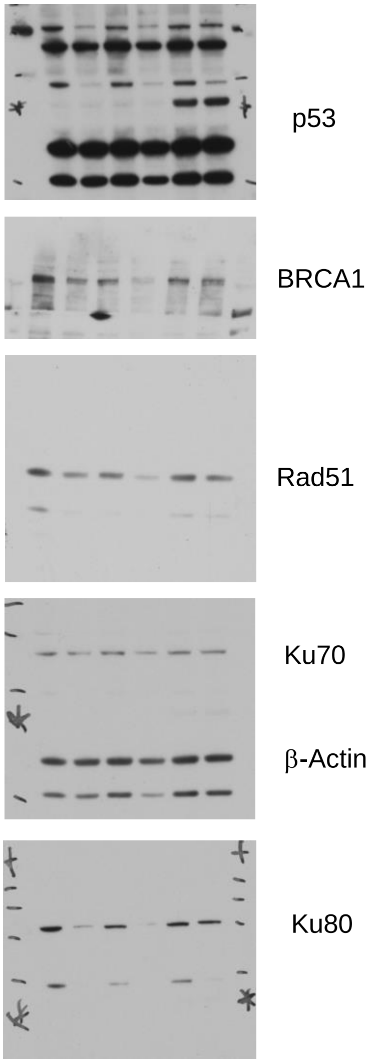

Fig.3A

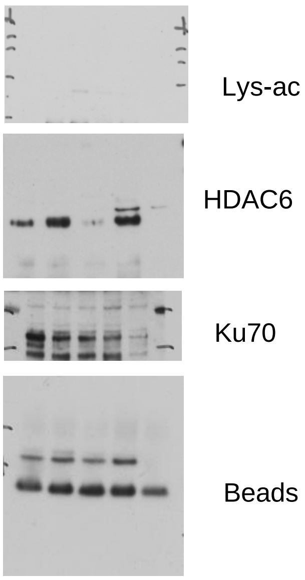

Fig.3D

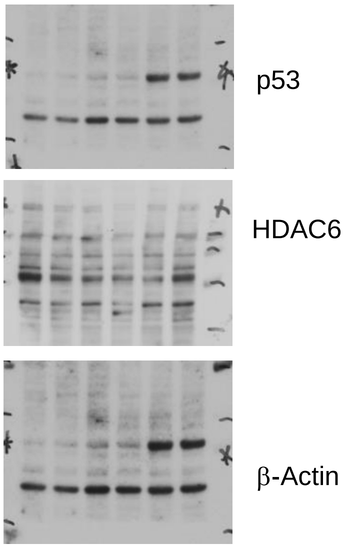

Fig.3F

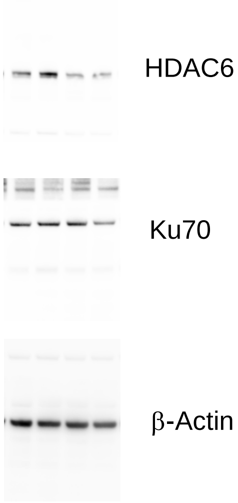

Fig.3B

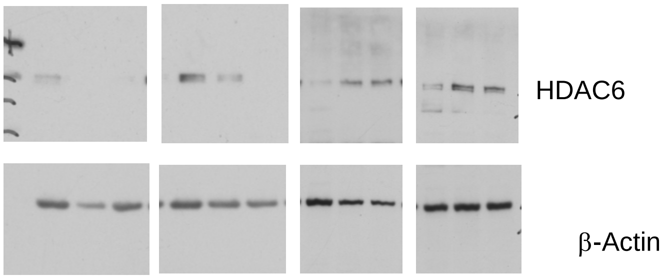

Fig.3G

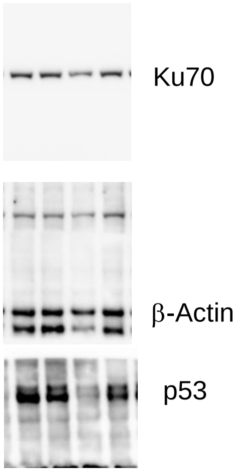

Fig.3H

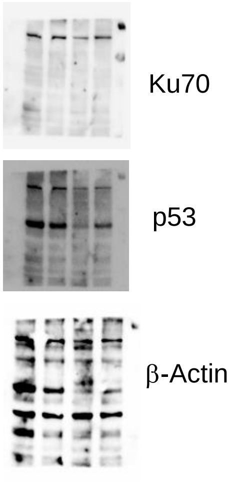

Fig.3C

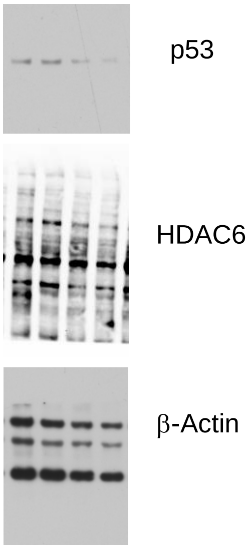

Fig.3E

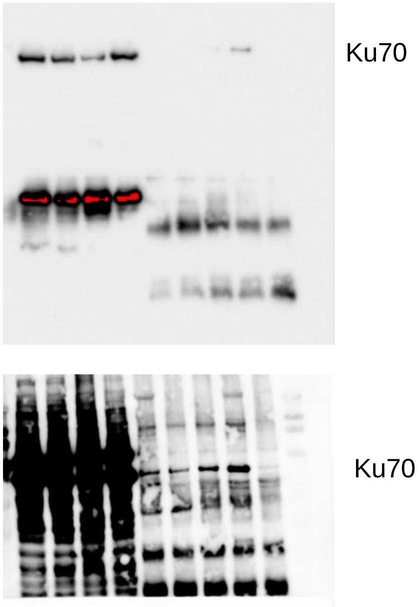

Fig.3I

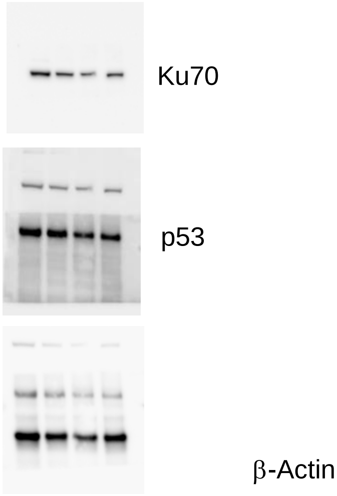

Fig.4A

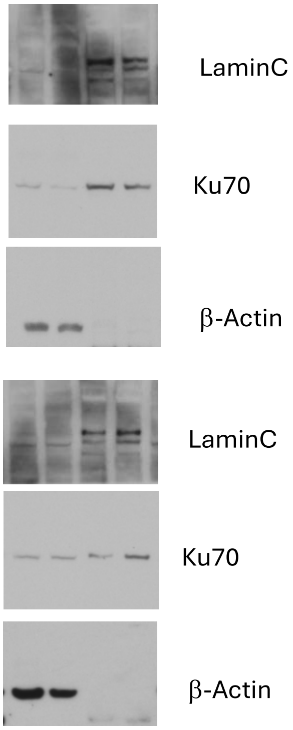

Fig.4D

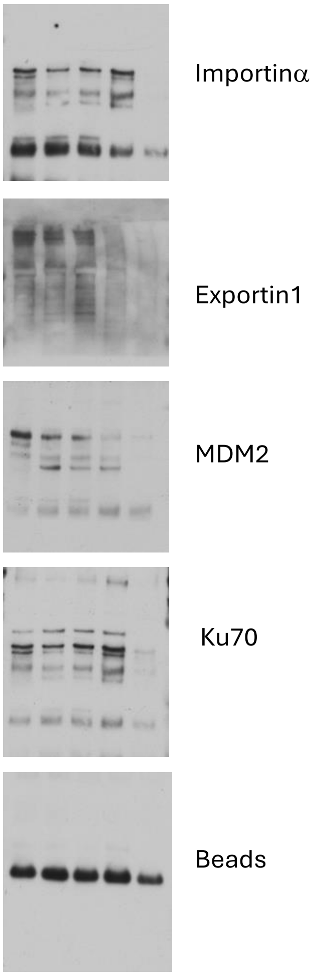

Fig.4E

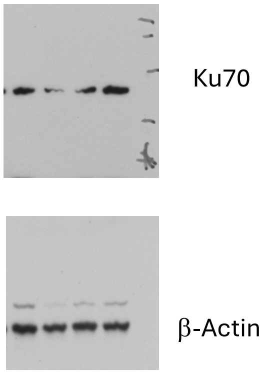

Fig.4F

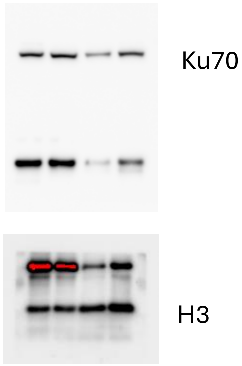

Fig.4C

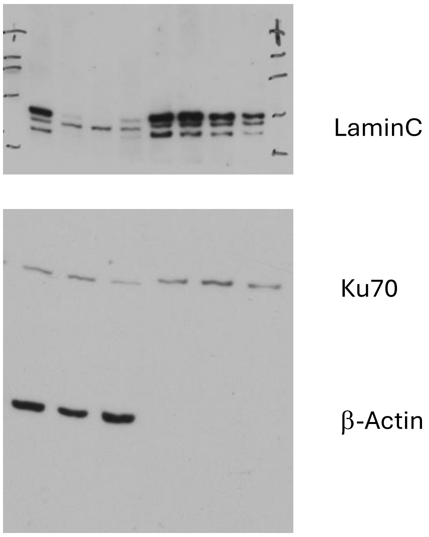

Fig.5A

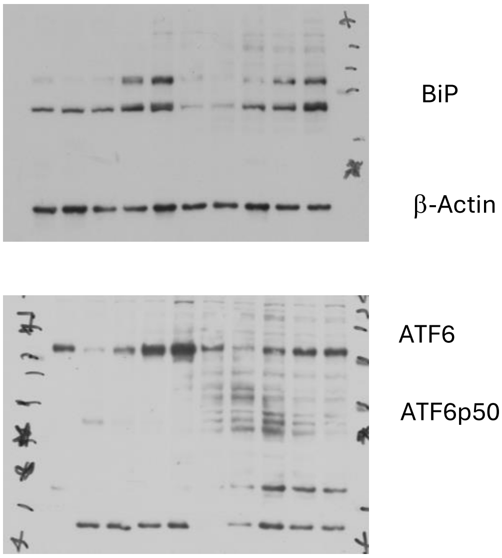

Fig.5B

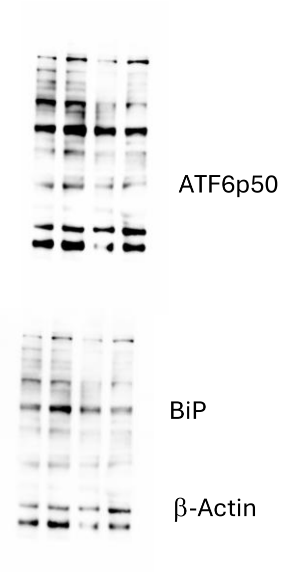

Fig.5C

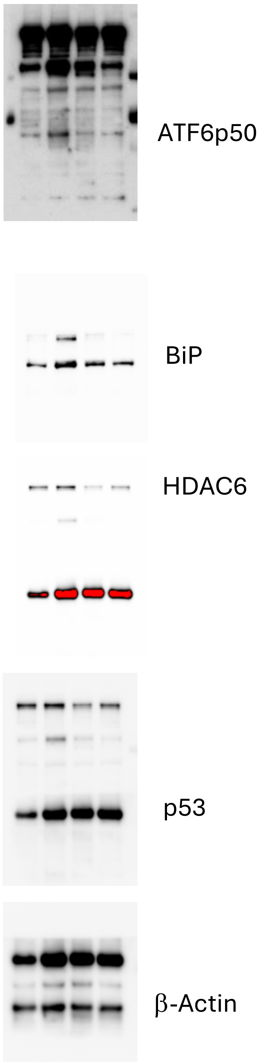

Fig.5D

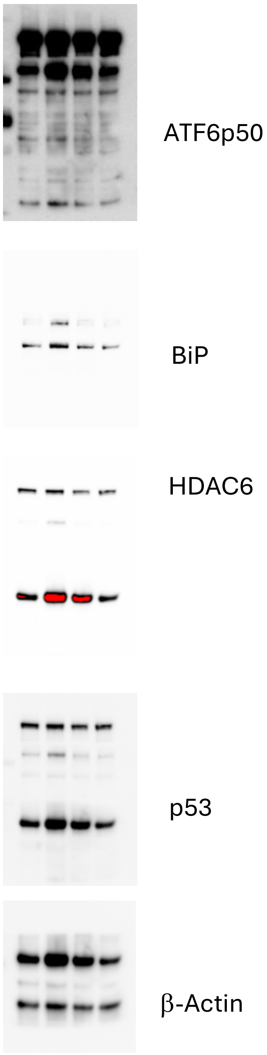

S2 B

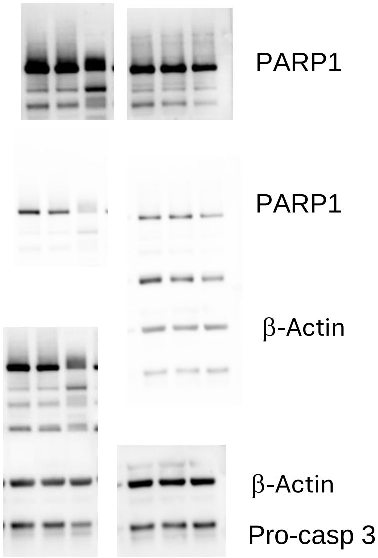

S4 A

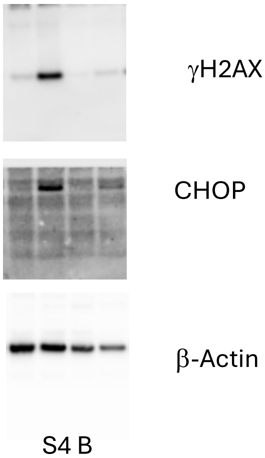

S4 B

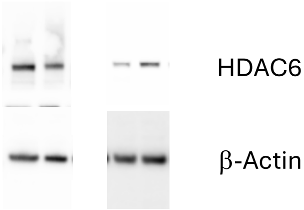

S4 C

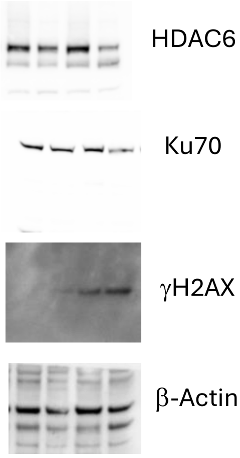

Supplement: Supplementary file 2 — Original blots [file 41420_2025_2433_MOESM2_ESM.pdf]
